# EDARA: An ERA5-based Dataset for Atmospheric River Analysis

**DOI:** 10.1038/s41597-024-03679-1

**Published:** 2024-08-17

**Authors:** Ruping Mo

**Affiliations:** National Laboratory-West, Meteorological Service of Canada, Environment and Climate Change Canada, Vancouver, BC Canada

**Keywords:** Atmospheric science, Environmental impact

## Abstract

Atmospheric Rivers (ARs) are long and narrow bands of strong horizontal water vapour transport concentrated in the lower troposphere. ARs play an important role in producing some high-impact weather events such as extreme precipitation and flooding, damaging winds, and temperature anomalies. To facilitate various studies on the short- and long-term variability of ARs and their impacts, I compiled a multi-decade global dataset containing 12 relevant meteorological variables for AR analysis. These variables were derived from the European Centre for Medium-Range Weather Forecasts atmospheric reanalysis version 5 (ERA5). They are available at 6-hour intervals from 1940 to present. Also included in the dataset is an interactive web browser-based graphical tool for visualising the AR evolution on regional (North America) and global scales. This ERA5-based Dataset for Atmospheric River Analysis (EDARA) may serve as a valuable resource for many AR-related research and applications.

## Background & Summary

**Fig. 1:**
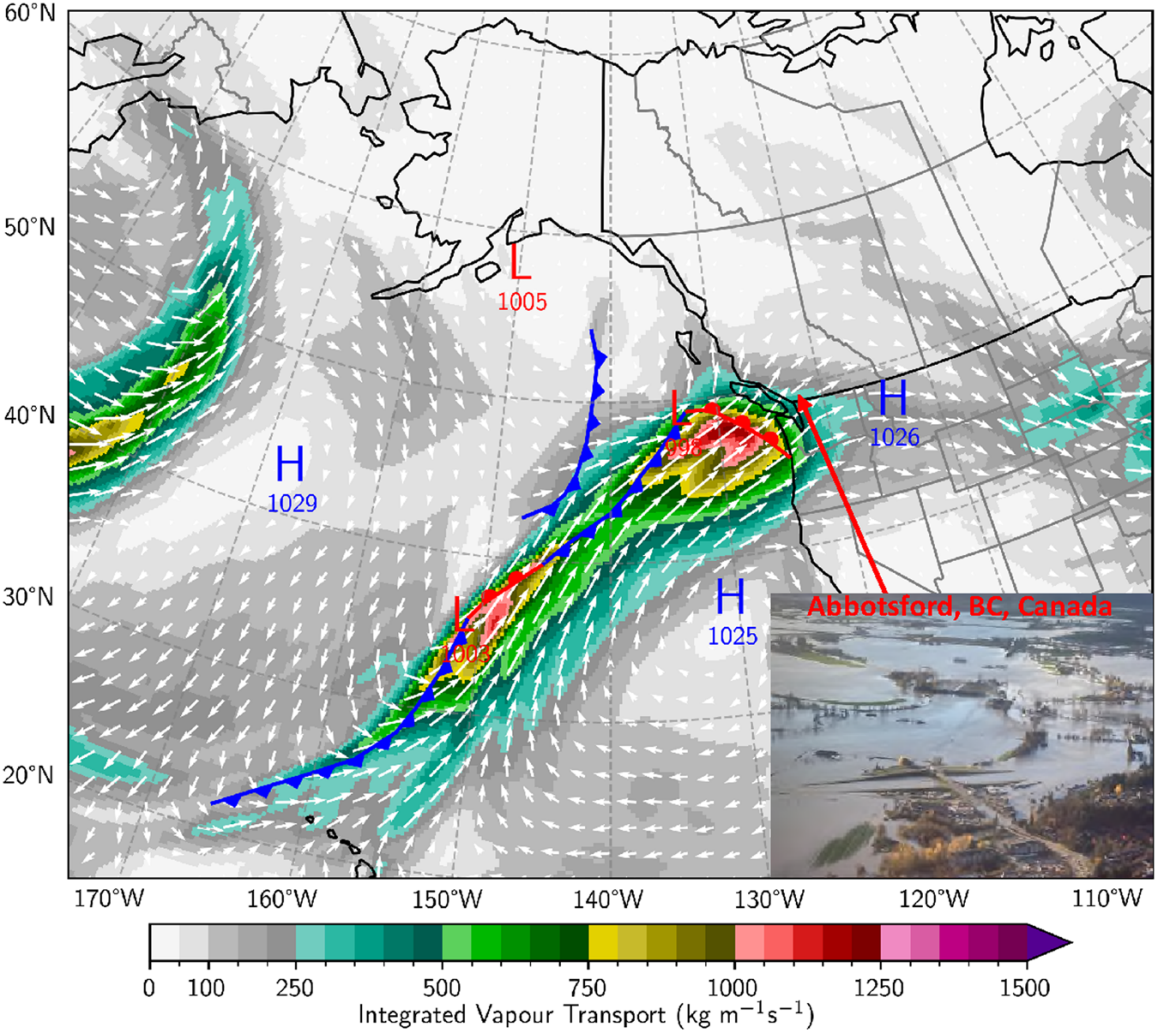
An atmospheric river making landfall on the west coast of North America at 0000 UTC 14 November 2021. The white arrow represents the vertically integrated water vapour flux and its magnitude is plotted as the integrated vapour transport (IVT) in coloured contours. Also plotted are cold fronts (blue lines with triangles), warm fronts (red lines with semicircles), cyclonic centres (red “L” with local minimum mean sea level pressure in hPa), anticyclonic centres (blue “H” with local maximum mean sea level pressure). The embedded photo is an aerial shot in the aftermath of the AR storm showing severe flood in Abbotsford, BC, Canada (courtesy of Abbotsford Police Department).

Atmospheric water vapour is critically important in the climate system. It not only serves as an infrared greenhouse blanket wrapped around the Earth, but also acts like weather fuel because of its capability to release latent heat energy upon condensation^1,2^. Atmospheric River (AR) is a weather phenomenon characterised by strong horizontal water vapour transport; it is typically associated with a low-level jet stream ahead of the cold front of an extratropical cyclone^3,4^. The river analogy was first conceived in the 1990s by a group of scientists who recognised the importance of some filamentary structures in atmospheric water vapour transport that often extend thousands of kilometres and may carry as much water as the Amazon River^5–8^. Similar features were also documented in earlier studies with various names such as “warm conveyor belt”^9–11^ or “moist tongue”^12,13^. Numerous studies in the past two decades have demonstrated the key roles of ARs in transporting moisture from the tropics to the mid-latitudes and triggering some high-impact weather events, such as extreme precipitation and flooding, damaging winds, and dangerous föhn or heatwave events^4,14–27^. As an example, Fig. 1 shows a strong AR making landfall on the west coast of North America at 0000 UTC 14 November 2021. This system stalled in this region for more than two days, producing extreme amounts of rainfall and causing catastrophic floods, landslides, and road washouts across southern British Columbia, Canada^28,29^. On the other hand, some AR events can be beneficial for providing critical water supply for ecosystems and human societies^17,30–32^.

Given their importance in shaping the global water cycle and potential in triggering high-impact weather events, ARs have emerged as a global focus of science and applications in recent years^33^. Understanding historical AR conditions and their future projections is critical for the global efforts on improving resilience against natural disasters in a changing climate. Analysing the spatiotemporal distribution of ARs requires three-dimensional meteorological data over multiple decades. Modern meteorological reanalyses, such as JRA-55^34^, MERRA-2^35^, and ERA5^36–39^, have been shown to perform reasonably well for tracking global AR activity^40–42^. However, the enormously large size of these datasets makes it generally impossible for many users to find desired information for their AR analyses. The purpose of this data descriptor is to present an ERA5-based Dataset for Atmospheric River Analysis (EDARA), which consists of global numerical data of 12 relevant variables and graphical AR catalogues at 6-hour intervals from 1940 to present. This compact dataset has been deposited to the Federated Research Data Repository (FRDR) at 10.20383/103.0935^43^. It can be partly downloaded for a specified month, and some AR features can be conveniently visualized online using an interactive web browser-based tool.

The two main approaches for tracking ARs involve analysing the vertically Integrated water Vapour Transport (IVT)^5–8,16,44–46^ and/or the Integrated Water Vapour (IWV)^4,15,17,18,47^. IVT combines and summarises moisture and wind profiles into an intensity measure of the overall horizontal water vapour transport, making it the most popular variable in AR analysis. IWV is a measure of total water content in vapour phase in the air column. It is also known as precipitable water vapour and can be used as a proxy for tracking ARs^4^. Based mainly on the spatiotemporal distribution of IVT or IWV, researchers have developed various AR identification methods with a wide range of considerations and conclusions^4,41,46–53^. The Atmospheric River Tracking Method Intercomparison Project (ARTMIP)^41,54,55^ is an international collaborative effort to understand and quantify the uncertainties in AR science that arise due to differences in identification methods. To facilitate method comparison, ARTMIP requires all participants to run their algorithms on a common reanalysis dataset and adhere to a common data format. The selected reanalysis is MERRA-2, which provides global meteorological data at an approximate horizontal resolution of 50 km at a 3-hour interval beginning in 1980^35^. The ARTMIP Tier-1 AR catalogues, together with derived variables (e.g., IVT and IWV) required for the algorithms, are now available from the Climate Data Gateway at 10.5065/D6R78D1M.

EDARA can be viewed as complementary to ARTMIP for AR analysis. ARTMIP is intended for uncertainty quantification associated with different AR detection algorithms. The main purpose of EDARA is to provide a large selection of relevant meteorological fields for in-depth AR analysis and model development (including machine learning approaches). As compared to MERRA-2, ERA5 has higher horizontal resolution (31 km) and covers a longer time span (recently extended back to 1940). In comparison to MERRA-2 and JRA-55, a recent study^42^ found that ERA5 has the smallest error in representing AR features observed using dropsondes. In addition to IWV and the two components (eastward and northward) of IVT, EDARA also includes nine supplemental variables to facilitate AR analysis. They are Column Relative Humidity (CRH), Total Precipitation Rate (TPR), 6-hour Total Precipitation (TP6H), 10 metre Gusty Wind Speed (GWS10m), Mean Sea Level Pressure (MSLP), 2 metre Temperature (T2m), Lower Tropospheric Mean Temperature (LTMT) based on the thickness between 500 and 1000 hPa, AR Shape (ARS) detected by an AR tracking algorithm developed by Guan *et al*.^49–51^, and a modified version of ARS (MARS); see Methods for detailed descriptions of these variables and the procedures used to derive or extract them from ERA5. With these global 6-hourly variables bundled together, EDARA can provide a unique one-stop point of data access for a wide range of AR research efforts, including ARTMIP-like tracking method comparisons, high-impact weather case studies, intraseasonal-to-interdecadal variability and climate change assessments, and machine learning model development. Furthermore, the potential uses of these multi-decade global meteorological variables go beyond AR analysis alone; they can also be used to investigate problems such as the comparison between ARs and non-ARs in general, or totally different phenomena.

## Methods

### The original reanalysis: ERA5

Modern meteorological reanalyses aim to provide a best estimate of the evolution of the atmospheric state by combining vast amounts of historical observations using advanced modelling and data assimilation systems^56^. ERA5 is the fifth-generation atmospheric reanalysis produced by the European Centre for Medium-Range Weather Forecasts (ECMWF)^36^. It provides hourly estimates of numerous atmospheric, land and oceanic climate variables from 1940 onwards, covering the Earth on a 31-km horizontal grid and resolving the atmosphere with 137 levels from the surface up to a height of 80 km. This state-of-the-art global dataset is freely available from the Climate Data Store of the Copernicus Climate Change Service (https://cds.climate.copernicus.eu/#!/home). It has been widely used in various meteorological and climatological studies, including recent applications in machine learning-based weather prediction^57,58^.

ERA5 pressure-level temperature, specific humidity, geopotential, eastward and northward wind components, together with single-level variables of surface pressure, total precipitation rate and amount, 10 metre wind components, and 2 metre temperature, are downloaded to produce the 6-hourly EDARA variables valid at 0000, 0600, 1200, and 1800 UTC.

### EDARA variables derived from ERA5

EDARA is an ERA5-based sub-dataset intended to support various AR analyses. It has been known that ARs are typically associated with a low-level jet stream ahead of the cold front of an extratropical cyclone^4,7^. Many meteorological fields available in ERA5 may be required for a comprehensive synoptic analysis of low-pressure and frontal systems. To keep the size of the dataset manageable, EDARA includes only the MSLP and T2m fields to fulfil this need. They are directly extracted from ERA5. T2m is also useful for investigating the temperature anomalies associated with AR activity^23,25–27^.

The two most important variables for AR analysis are IVT and IWV. ERA5 provides a quantity called the total column water vapour, which can be used as IWV. Also available in ERA5 are the vertical integrals of eastward and northward water vapour flux, which can be used as the two vector components of IVT. These total-column quantities are based on integrating from the Earth’s surface to the top of the atmosphere. However, given that reliable radiosonde measurements are limited to within the troposphere, the IVT and IWV fields used in many AR studies were calculated from vertical integrations from near-surface up to a pressure level between 300 and 100 hPa^5–8,41,45–49^. In keeping with this tradition, the IWV and IVT in EDARA are calculated from the following equations using the ERA5 moisture and wind profiles,

1 $${\rm{I}}{\rm{W}}{\rm{V}}={\int }_{0}^{{z}_{{\rm{t}}{\rm{o}}{\rm{p}}}}\rho q\,{\rm{d}}z=\frac{1}{g}{\int }_{{p}_{{\rm{t}}{\rm{o}}{\rm{p}}}}^{{p}_{{\rm{s}}{\rm{f}}{\rm{c}}}}q\,{\rm{d}}p$$

2 $${\rm{I}}{\rm{V}}{\rm{T}}=\sqrt{{Q}_{u}^{2}+{Q}_{v}^{2}},\,\,\,\,({Q}_{u},{Q}_{v})={\int }_{0}^{{z}_{{\rm{t}}{\rm{o}}{\rm{p}}}}(u,v)\rho q\,{\rm{d}}z=\frac{1}{g}{\int }_{{p}_{{\rm{t}}{\rm{o}}{\rm{p}}}}^{{p}_{{\rm{s}}{\rm{f}}{\rm{c}}}}(u,v)q\,{\rm{d}}p$$

where $\rho$ is moist air density, $q$ is specific humidity, $u$ and $v$ are eastward and northward wind components, ${Q}_{u}$ and ${Q}_{v}$ represent eastward and northward components of integrated water vapour flux, $g=9.80665\,{\rm{m}}\,{{\rm{s}}}^{-2}$ is the gravitational acceleration, $z$ is upward distance from the Earth surface, and $p$ is air pressure. The change of vertical coordinate from $z$ to $p$ in the above equations is based on the hydrostatic equation (${\rm{d}}p/{\rm{d}}z=-\rho g$)^59^. For EDARA, the vertical integration is from the surface $(z=0,p={p}_{{\rm{sfc}}})$ to a level near the tropopause at $p={p}_{{\rm{top}}}=200$ hPa. Since specific humidity decreases rapidly with increasing altitude in the atmosphere, changing ${p}_{{\rm{top}}}$ to a different value in the range from 300 to 100 hPa has negligible impact on the above integrations^60^.

**Fig. 2:**
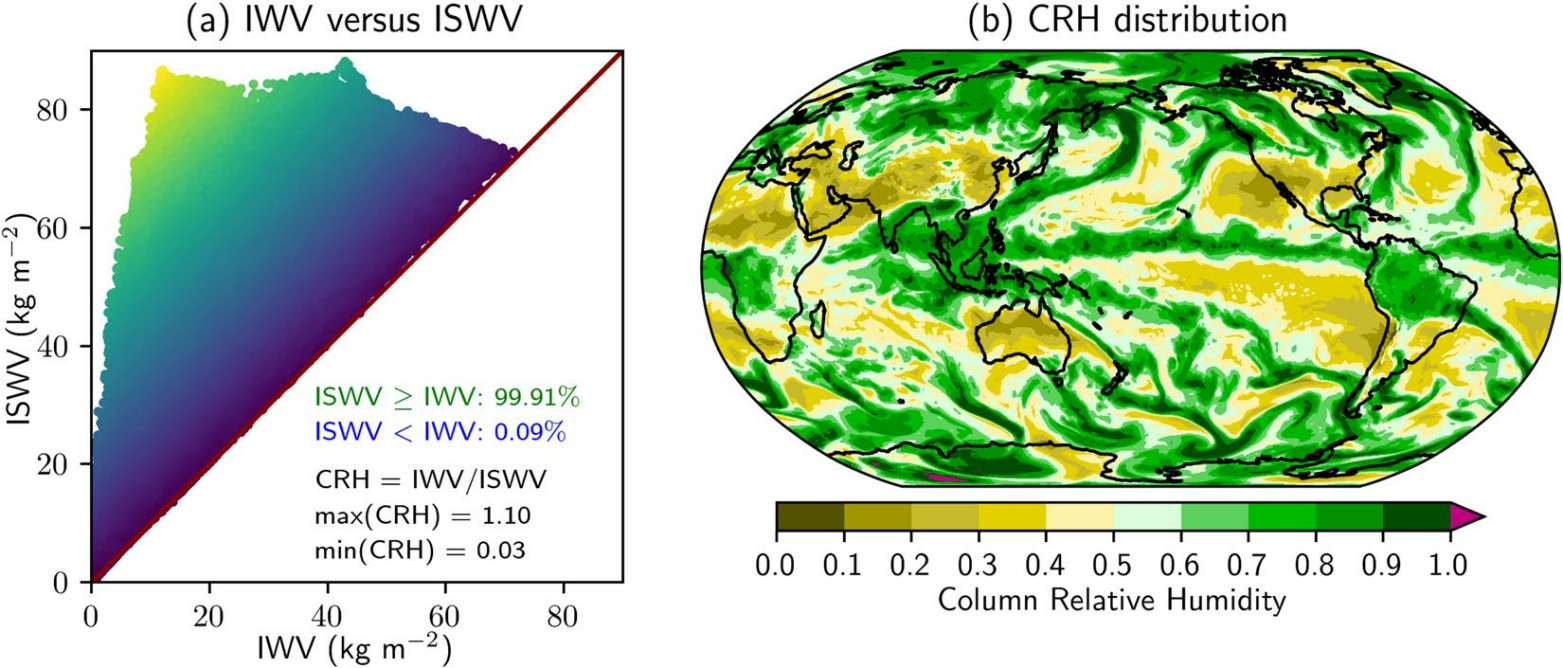
(**a**) Comparison of the integrated water vapour (IWV) with its saturation counterpart (ISWV) at all grid points over the global domain, valid at 0000 UTC 14 November 2021. (**b**) The corresponding distribution of column relative humidity (CRH = IWV/ISWV) showing some areas over the Antarctic where CRH > 1.

A useful supplementary field for assessing the AR-induced precipitation potential is the column relative humidity (CRH), which can be defined as the ratio of IWV to the integrated saturation water vapour (ISWV)^60^,

3 $${\rm{C}}{\rm{R}}{\rm{H}}=\frac{{\rm{I}}{\rm{W}}{\rm{V}}}{{\rm{I}}{\rm{S}}{\rm{W}}{\rm{V}}},\,\,\,\,{\rm{I}}{\rm{S}}{\rm{W}}{\rm{V}}=\frac{1}{g}{\int }_{{p}_{{\rm{t}}{\rm{o}}{\rm{p}}}}^{{p}_{{\rm{s}}{\rm{f}}{\rm{c}}}}{q}_{{\rm{s}}}\,{\rm{d}}p$$

where ${q}_{{\rm{s}}}$ is the specific humidity of the atmosphere when it is saturated. Although it is generally true that $0\le {\rm{CRH}}\le 1$, there are rare occasions when a supersaturated state (i.e., ${q}_{s} < q$) can be reached in the atmosphere^61,62^, giving the potential for ${\rm{CRH}} > 1$. Figure 2 shows a case valid at 0000 UTC 14 November 2021, in which some small areas (less than 1% of the total grid points) with ${\rm{CRH}} > 1$ can be seen over the Antarctic. These anomalies could be either related to the supersaturated conditions or computational error. The formula for ${q}_{{\rm{s}}}$ used to calculate CRH in EDARA can be found in Mo *et al*.^60^.

ARs have the potential to produce heavy precipitation events upon landfall. The TPR variable in EDARA can be used to assess precipitation associated with ARs. It is the sum of the four precipitation intensity parameters in ERA5: the rates of convective and large-scale rain and snowfall water equivalent. The units given in ERA5 for these four precipitation rates are ${\rm{k}}{\rm{g}}\,{{\rm{m}}}^{-2}{{\rm{s}}}^{-1}$. They are equivalent to ${\rm{m}}{\rm{m}}\,{{\rm{s}}}^{-1}$, since 1 kg of water spreading over 1 square metre of surface is 1 mm deep (neglecting the effects of temperature on the density of water)^37^. The units for TPR in EDARA are given as ${\rm{m}}{\rm{m}}\,{{\rm{h}}}^{-1}$, which is more commonly used in practice. Note that 1 ${\rm{m}}{\rm{m}}\,{{\rm{s}}}^{-1}$ = 3600 ${\rm{m}}{\rm{m}}\,{{\rm{h}}}^{-1}$; the larger data values can be more efficiently stored in a compressed netCDF format. Another precipitation variable included in EDARA is TP6H, which is the sum of the ERA5 hourly total precipitation over the last six hours. This parameter is useful for estimating the storm-total precipitation during an AR event.

**Fig. 3:**
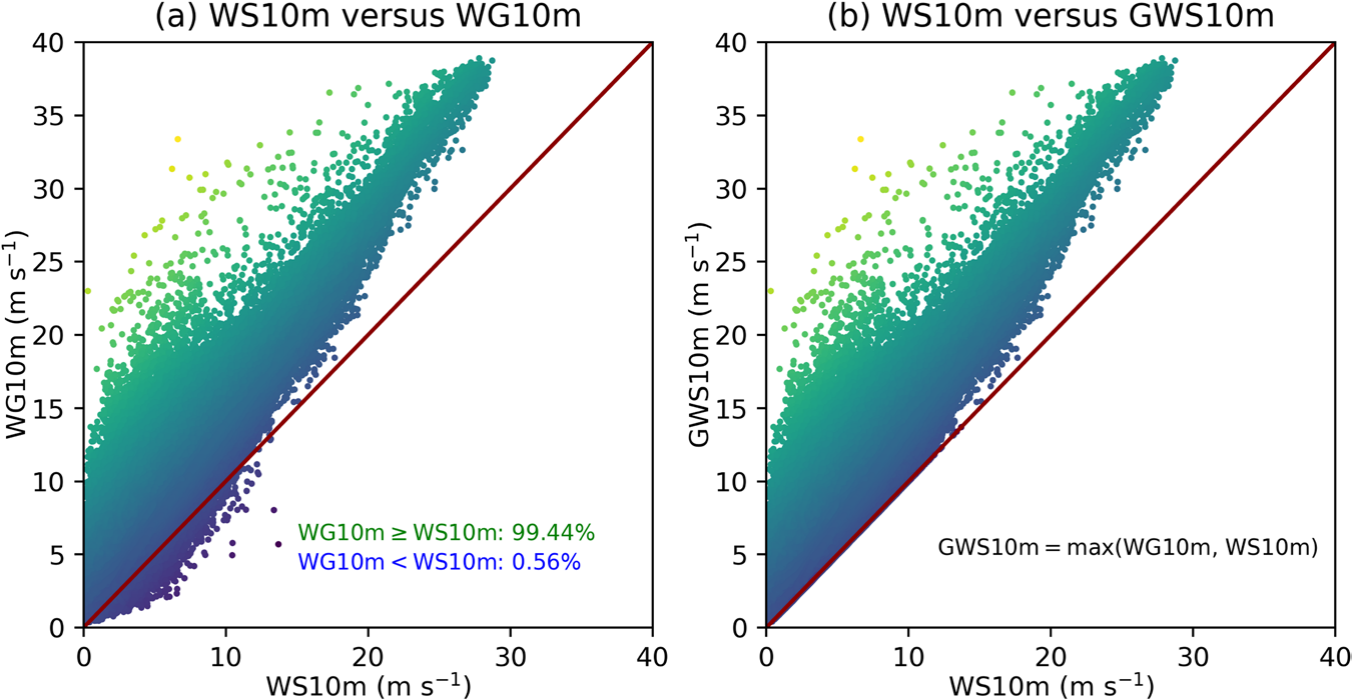
(**a**) Comparison of the ERA5 10 metre wind speed (WS10m) with the 10 metre wind gust (WG10m) at all grid points over the global domain, valid at 0000 UTC 14 November 2021. (**b**) The corresponding comparison of the ERA5 WG10m with the EDARA 10 metre gusty wind speed (GWS10m).

Landfalling ARs also have the potential to produce extreme wind events^22^. This can be evaluated using the near-surface wind and gust speeds available in ERA5. According to the World Meteorological Organisation^63^, the surface wind speeds for standard weather reports are winds measured at 10 metre height averaged over the last 10 minutes (hereafter, WS10m), and the 10 metre gusts (WG10m) should be determined from 3-second running means. The ERA5 10 metre eastward ($u$) and northward ($v$) wind components at each grid point can be used to calculate WS10m (i.e., ${\rm{WS}}10{\rm{m}}=\sqrt{{u}^{2}+{v}^{2}}$). This quantity is not sufficient to capture the turbulent nature of wind fluctuations during extreme wind events. As WG10m is concerned, the ECMWF model for ERA5 cannot explicitly resolve eddies of all scales responsible for the near-surface gusts, and the 3-second average duration is shorter than a model time step. Therefore, the parametrised gusts in ERA5 are computed by some post-processing procedures that combine the analysed 10 metre wind speed with the turbulent gustiness and the convective contribution^64,65^. An ERA5 parameter representing the maximum 10 metre wind gust since previous post-processing (1 hour period) is taken as WG10m. It would be expected that ${\rm{WG}}10{\rm{m}}\ge {\rm{WS}}10{\rm{m}}$. However, it is a known issue of ERA5 that the analysed WS10m can be larger than the forecast WG10m^39^. Figure 3 shows an example valid at 0000 UTC 14 November 2021, in which about 0.6% of the total grid points have ${\rm{WG}}10{\rm{m}} < {\rm{WS}}10{\rm{m}}$. To avoid this apparent paradox, the GWS10m variable in EDARA is defined as the larger value between WS10m and WG10m, i.e.,

4 $${\rm{GWS}}10{\rm{m}}=\max \left({\rm{WS}}10{\rm{m}},{\rm{WG}}10{\rm{m}}\right)$$

ARs may be referred to as effective moisture conveyor belts in the atmosphere^66^. They are closely related to the concept of warm conveyor belt and can play a key role in heat transport in the lower troposphere^11,25–27,66^. To assess their contribution to the temperature distribution and the potential to trigger extreme heat wave events, EDARA not only includes the T2m field from ERA5, but also derives a lower tropospheric mean temperature from the thickness between 1000 and 500 hPa. In general, a mean temperature $\widetilde{T}$ for a layer between two pressure levels ${p}_{0}$ and ${p}_{1}$ can be defined as^59^

5 $$\widetilde{T}=\frac{g({Z}_{1}-{Z}_{0})}{R(\mathrm{ln}{p}_{0}-\mathrm{ln}{p}_{1})}=\frac{g{Z}_{T}}{R\,\mathrm{ln}({p}_{0}/{p}_{1})}$$

where $R=287\,{\rm{J}}\,{{\rm{K}}}^{-1}{\rm{k}}{{\rm{g}}}^{-1}$ is the gas constant for dry air, ${Z}_{0}$ and ${Z}_{1}$ are the geopotential heights (in metres) at ${p}_{0}$ and ${p}_{1}$, respectively, and ${Z}_{T}={Z}_{1}-{Z}_{0}$ is the thickness of the layer. For ${p}_{0}=1000$ hPa and ${p}_{1}=500$ hPa, the LTMT (in K) in EDARA is computed from $\mathop{T}\limits^{ \sim }=g{[R{\rm{l}}{\rm{n}}(2)]}^{-1}\times {Z}_{T}\cong 0.0493\times {Z}_{T}$. On the other hand, if the thickness field is needed instead, it can be readily recovered from ${Z}_{T}={g}^{-1}R\,\mathrm{ln}(2)\times \widetilde{T}\cong 20.286\times \widetilde{T}$.

### Graphical AR catalogues

**Fig. 4:**
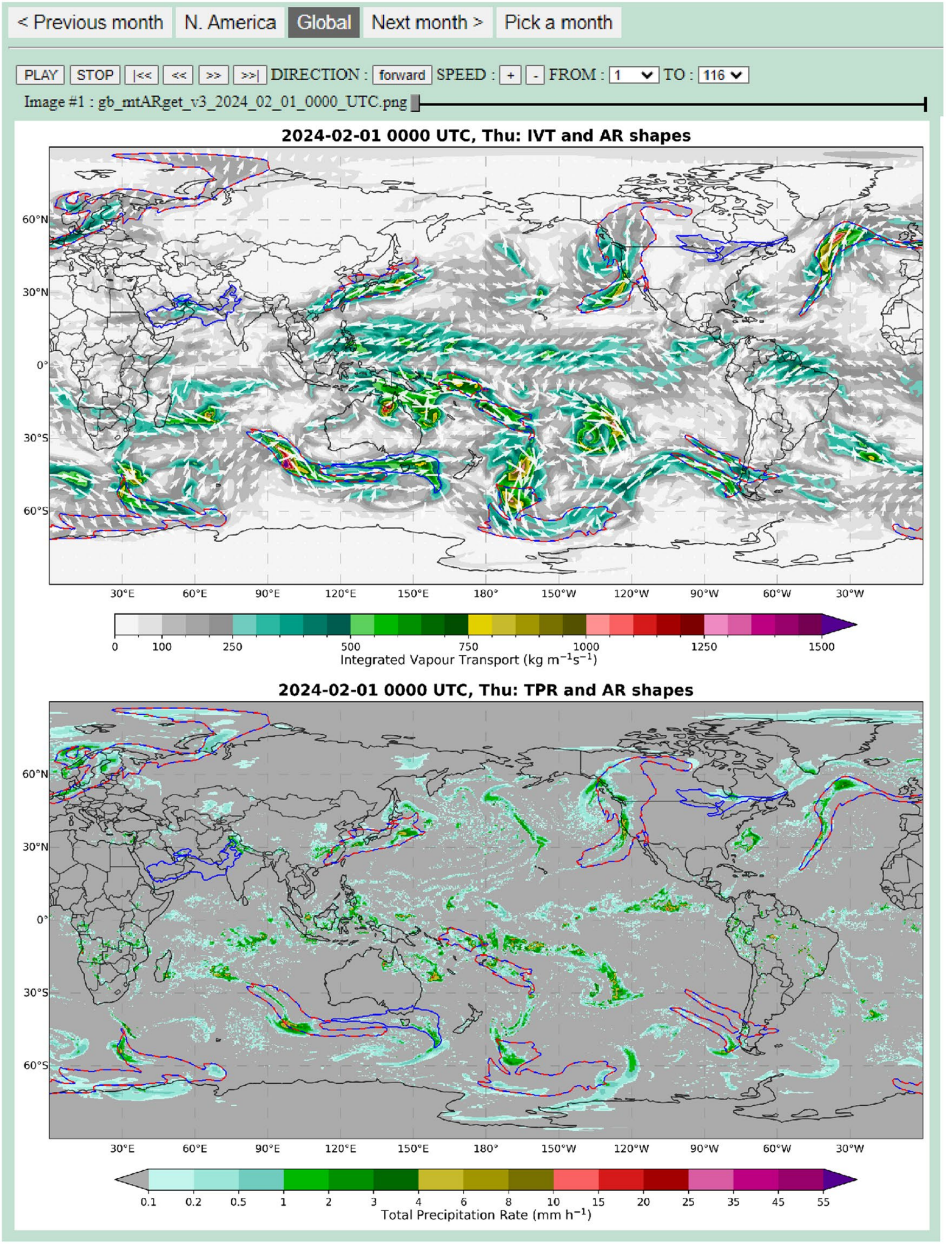
The interface of graphical AR catalogue for February 2024 (Global Domain). The atmospheric river boundaries determined by the tARget-v3 and mtARget-v3 algorithms are marked on the maps by red dashed lines and blue solid lines, respectively. The top panel also shows the IVT distribution and its vector representation (Qu, Qv). The TPR distribution is shown in the bottom panel. Users can switch to a zoomed-in domain (see Fig. 5) by clicking the “N. America” button. Convenient settings are available for running the animation through the whole month at a 6-hourly time step.

**Fig. 5:**
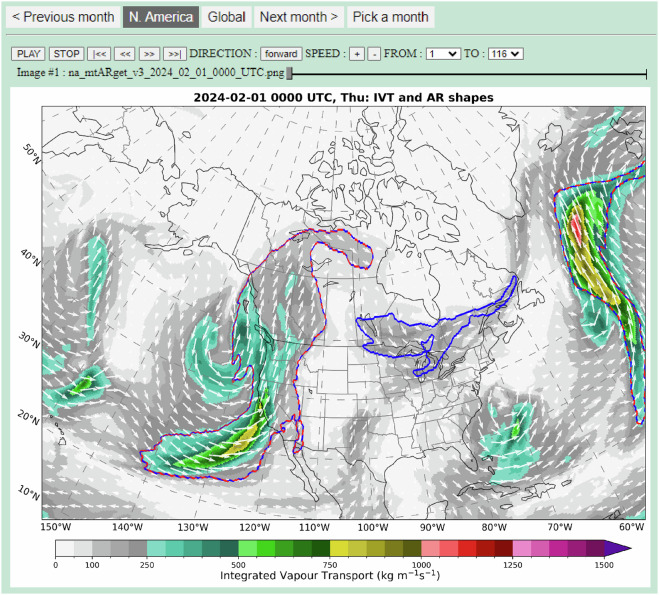
The interface of graphical AR catalogue for February 2024 (North American Domain). The screenshot depicts an atmospheric river affecting the west coast of the United States at 0000 UTC 1 February 2024. The atmospheric river boundaries determined by the tARget-v3 and mtARget-v3 algorithms are marked on the maps by red dashed lines and blue solid lines, respectively. The IVT distribution and its vector representation (Qu, Qv) are also plotted on the map.

EDARA is not just a numerical database. It also includes a graphical tool for visualising AR features and their evolution. The tool can be accessed using any standard web browser. The monthly graphical AR catalogue consists of a global view (Fig. 4) and a zoomed-in view over North America (Fig. 5). For the global domain, the boundaries of AR shapes (ARS, red dashed lines) and the modified AR shapes (MARS, blue solid lines) are plotted over the IVT and TPR maps, respectively; only the IVT-ARS/MARS map is available for the North American domain. The ARS field is computed using the Tracking Atmospheric Rivers Globally as Elongated Targets, version 3 (tARget-v3) algorithm developed by Guan and Waliser^51^. The MARS field is computed using a modified version of tARget-v3 (denoted as mtARget-v3). Note that the ARS contours (red dashed lines) were plotted on top of the MARS contours (blue solid lines). Therefore, where the ARS and MARS contours are co-located, they are represented by the red-blue lines.

In a nutshell, the tARget-v3 algorithm uses a combination of IVT geometry and intensity thresholds to identify ARs at a given time^51^. It first extracts contiguous areas of connected pixels based on IVT exceeding the magnitude threshold, which is a combination of a given percentile and a fixed lower limit. The lower limit is set at 100 kg m^−1^ s^−1^ and the initial percentile is at the 85th. Requirements on the direction of mean IVT (poleward component > 50 kg m^−1^ s^−1^), coherence of IVT directions (more than half of the area having an IVT direction within 45° from the mean IVT), length (>2,000 km) and length/width ratio (>2) are then applied to these IVT objects. The requirement for the mean IVT direction is to comply with the notion that ARs transport moisture from low to high latitudes^8,49^. IVT objects failing the above requirements are subject to a series of iterations where the threshold on IVT magnitude is first raised to the 87.5th percentile and then, if necessary, to the 90th, 92.5th, and 95th percentile. Each identified AR object is assigned a unique numerical ID (i.e., 1, 2, …), and the ID number is populated to all grid cells within the boundary that forms the corresponding object.

**Fig. 6:**
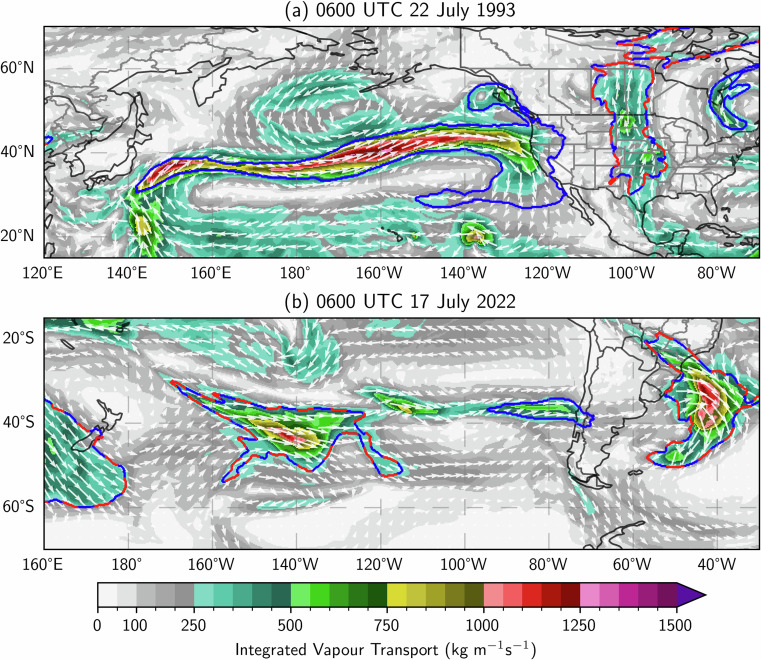
Distributions of IVT (Qu, Qv), ARS (red dashed lines) and MARS (blue solid lines) valid at (**a**) 0600 UTC 22 July 1993 and (**b**) 0600 UTC 17 July 2022, respectively.

The tARget-v3 algorithm^51^ is used to derive the ARS variable in EDARA. The MATLAB code for this algorithm was provided by Dr. Bin Guan. For each of the 12 months, the IVT percentiles are calculated over all 6-hourly time steps during the 5 months centred on that month over the period of 1991–2020. The value of the ARS variable is given as either 0 for the absence of AR or 1 for the presence of AR. The same rules apply to the mtARget-v3 algorithm (and the MARS variable), except that the requirement on the direction of mean IVT (poleward component > 50 kg m^−1^ s^−1^) is applied only over the tropical region between 20°S and 20°N. This modification allows some zonally oriented or equatorward-moving objects in the extratropical areas to be identified as ARs. Figure 6a shows an example valid at 0600 UTC 22 July 1993. A zonally oriented object of strong IVT across the North Pacific is identified as an AR by the mtARget-v3 algorithm (blue solid lines), but not by the original tARget-v3 that applies the mean IVT direction requirement globally. This warm-season AR may be called an “oriental express”, given that its origin can be traced to the Southeast Asian summer monsoon^26^. In addition, a northerly moist flow from the cyclonic wrap-around over Eastern Canada is also identified as an AR object by the mtARget-v3 algorithm (Fig. 6a). Figure 6b shows another zonal AR making landfall in south-central Chile at 0600 UTC 17 July 2022. This event was investigated in a recent study^67^. Note that the issue of zonal ARs was also raised by Pan and Lu^53^. Furthermore, during the review process of this article, it was brought to the author’s attention that the fourth version of the tARget algorithm (tARget-v4) had been published, in which some further refinements were introduced to identify zonal ARs in the extratropical atmosphere^68^.

Outputs of the tARget-v3 and mtARget-v3 algorithms include the AR shape, axis, landfall location, and basic statistics of each detected AR object; only the AR shape fields are used to derive the ARS and MARS variables in the EDARA suite. These two variables, ARS and MARS, can be compared with their counterparts derived from the tARget-v4 algorithm^68^ or other methods, such as PanLu2.0^53,69^.

## Data Records

The dataset (EDARA) is available at FRDR (https://www.frdr-dfdr.ca/repo) and can be accessed at 10.20383/103.0935^43^. It consists of two components: the numerical data and the graphical AR catalogues. This section is the primary source of information on the availability and content of the data being described.

### Numerical data

The numerical data component of EDARA consists of monthly netCDF files from 1940 to present. These data files, named in the form era5dara_yyyymm.nc (e.g., era5dara_202312.nc), can be found in the “data” folder. In each file, 6-hourly (0000, 0600, 1200, 1800 UTC) data of 12 variables through a month are stored. The spatial resolution is a 0.25° global grid. The 12 variables in the dataset are summarised in Table 1.

**Table 1:** Variables included in EDARA.

Variables	Abbreviations	Units	Notes
Mean sea level pressure	MSLP	Pa	Extracted directly from ERA5.
Integrated water vapour	IWV	${\rm{k}}{\rm{g}}\,{{\rm{m}}}^{-2}$ (*or* mm)	Computed by Eq. (1) with integration up to 200 hPa.
Integrated eastward water vapour flux	Qu	${\rm{k}}{\rm{g}}\,{{\rm{m}}}^{-1}{{\rm{s}}}^{-1}$	Computed by Eq. (2) with integration up to 200 hPa.
Integrated northward water vapour flux	Qv	${\rm{k}}{\rm{g}}\,{{\rm{m}}}^{-1}{s}^{-1}$	Computed by Eq. (2) with integration up to 200 hPa.
Column relative humidity	CRH	none	Computed by Eq. (3) with integration up to 200 hPa.
Total precipitation rate	TPR	${\rm{k}}{\rm{g}}\,{{\rm{m}}}^{-2}{{\rm{h}}}^{-1}$ $(or\,{\rm{m}}{\rm{m}}\,{{\rm{h}}}^{-1})$	Sum of four precipitation rates in ERA5.
6-hour total precipitation	TP6H	mm	Sum of total precipitation over the last six hours in ERA5.
Gusty wind speed at 10-m height	GWS10m	${\rm{m}}\,{{\rm{s}}}^{-1}$	Combined wind speed computed by Eq. (4).
Temperature at 2-metre height	T2m	K	Extracted directly from ERA5.
Lower-tropospheric mean temperature	LTMT	K	Thickness-based quantity computed by Eq. (5).
AR shape (0: non-AR and 1: AR)	ARS	none	Calculated by the tARget-v3 algorithm; value = 0 or 1.
Modified AR shape (0: non-AR and 1: AR)	MARS	none	Calculated by the mtARget-v3 algorithm; value = 0 or 1.

### Graphical AR catalogues

Monthly graphical AR catalogues can be accessed via any standard web browser in the “figs” folder. For each calendar month, there is a folder containing an HTML file named index.html and two subfolders “gb” and “na” holding the AR images over the global (Fig. 4) and North American (Fig. 5) domains, respectively. Opening the index.html file with a browser brings up a map of the IVT, ARS (red dashed lines) and MARS (blue solid lines) distributions over the North American domain. There are forward and backward buttons for animation control. The two domain buttons allow users to switch between the North American and global views. The global view also shows the TPR distribution. The 6-hourly images in the gb subfolder are named in the form gb_mtARget_yyyy_mm_dd_hh00_UTC.png, where yyyy, mm, dd and hh represent year, month, day, and hour, respectively. Similarly, the images in the na subfolder are named as na_mtARget_yyyy_mm_dd_hh00_UTC.png.

### Additional program and data files

The README file in the top-level directory provides detailed technical information to researchers about how to use this dataset. There are twelve additional program and data files located in the “misc” folder. They are included mainly for demonstration purposes. Descriptions of three program files are as follows:

1. misc/mtarget.m. This MATLAB program demonstrates how to track ARs globally using the tARget-v3 and mtARget-v3 algorithms (see Methods section above). It takes one key parameter and three data files as inputs and generates an output file. For key = 0 (or key = 1), the program executes the tARget-v3 (or mtARget-v3) algorithm.
2. misc/Derive_variables_from_ERA5.py. This python program is used to illustrate the computational procedure for deriving some EDARA variables from ERA5 data. It takes five data files as inputs.
3. misc/Extract_variables_from_era5dara.py. This python program illustrates how to access data in an EDARA numerical data file. It takes one data file as input.

The nine data files in the misc folder are either the input data for the above programs or the output data from them. For their descriptions, users can consult the README file from the top-level directory.

## Technical Validation

Technical validation of this dataset was mainly achieved by manually playing animations through all the graphical catalogues and comparing some historical high-impact weather events with the AR features documented in the catalogues. As shown in Fig. 1, the IVT (Qu, Qv) and frontal distributions provide a concise, visual representation of a landfalling AR that triggered the catastrophic floods in southern British Columbia in mid-November 2021^28,29^.

**Fig. 7:**
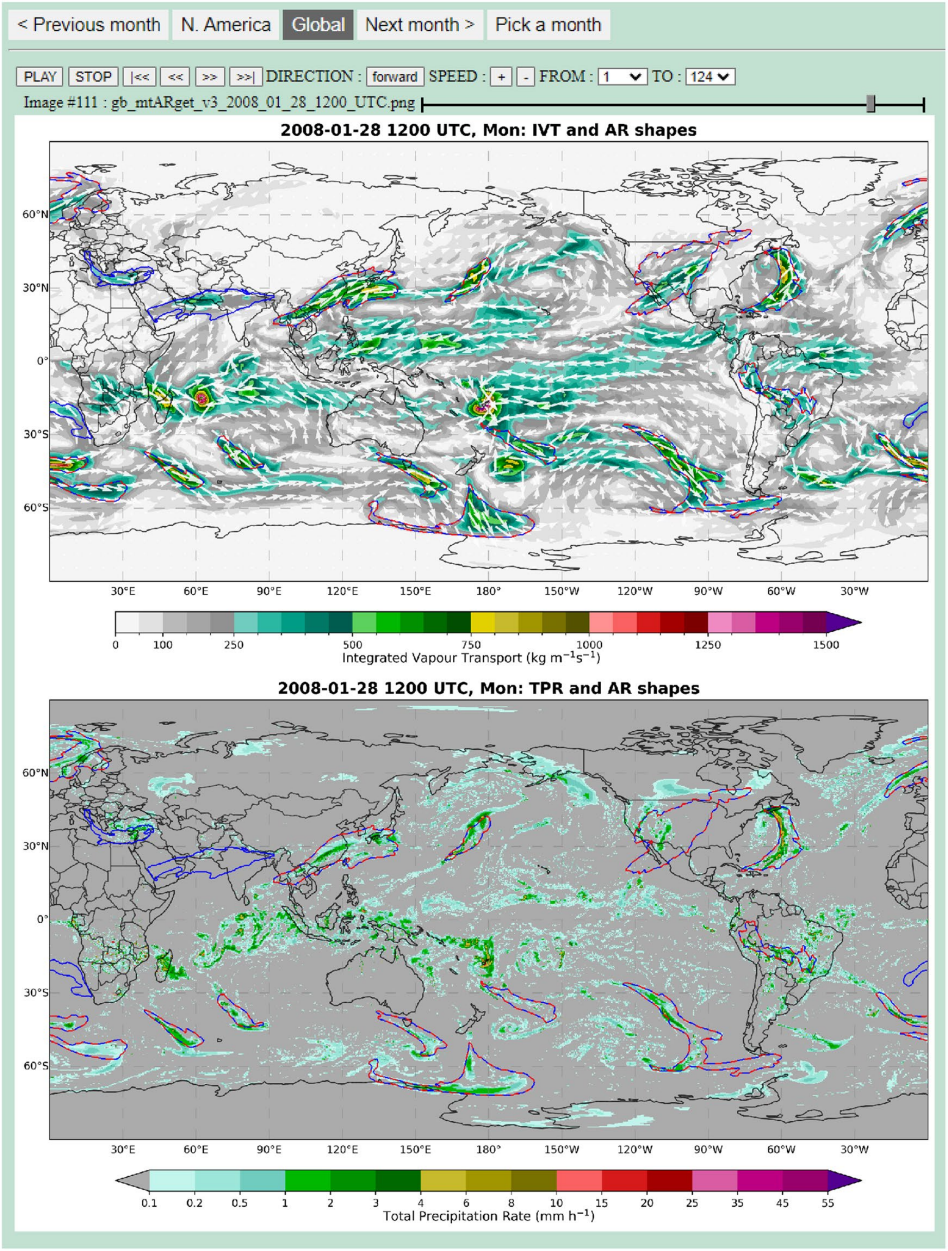
A screenshot of the graphical AR catalogue valid at 1200 UTC 28 January 2008 over the global domain.

Figure 5 shows a major AR affecting western United States at 0000 UTC 1 February 2024. It was one of the two early February ARs that brought extensive flooding, intense winds, power outages, and road closures to California^70^. Zooming out to the global domain (Fig. 4) brings up attention to an East Asian AR over the Northwest Pacific. Stepping back to the catalogue of the previous month shows that this AR began to develop over the central and eastern regions of China on 30 January. It made a significant contribution to the most complex winter weather conditions during the Lunar New Year travel rush in China since 2008^71^. As a reference to the great 2008 Chinese ice storm^72^, users can use the graphical tool to visualize the AR development from 24 January to 2 February (e.g. Fig. 7).

**Fig. 8:**
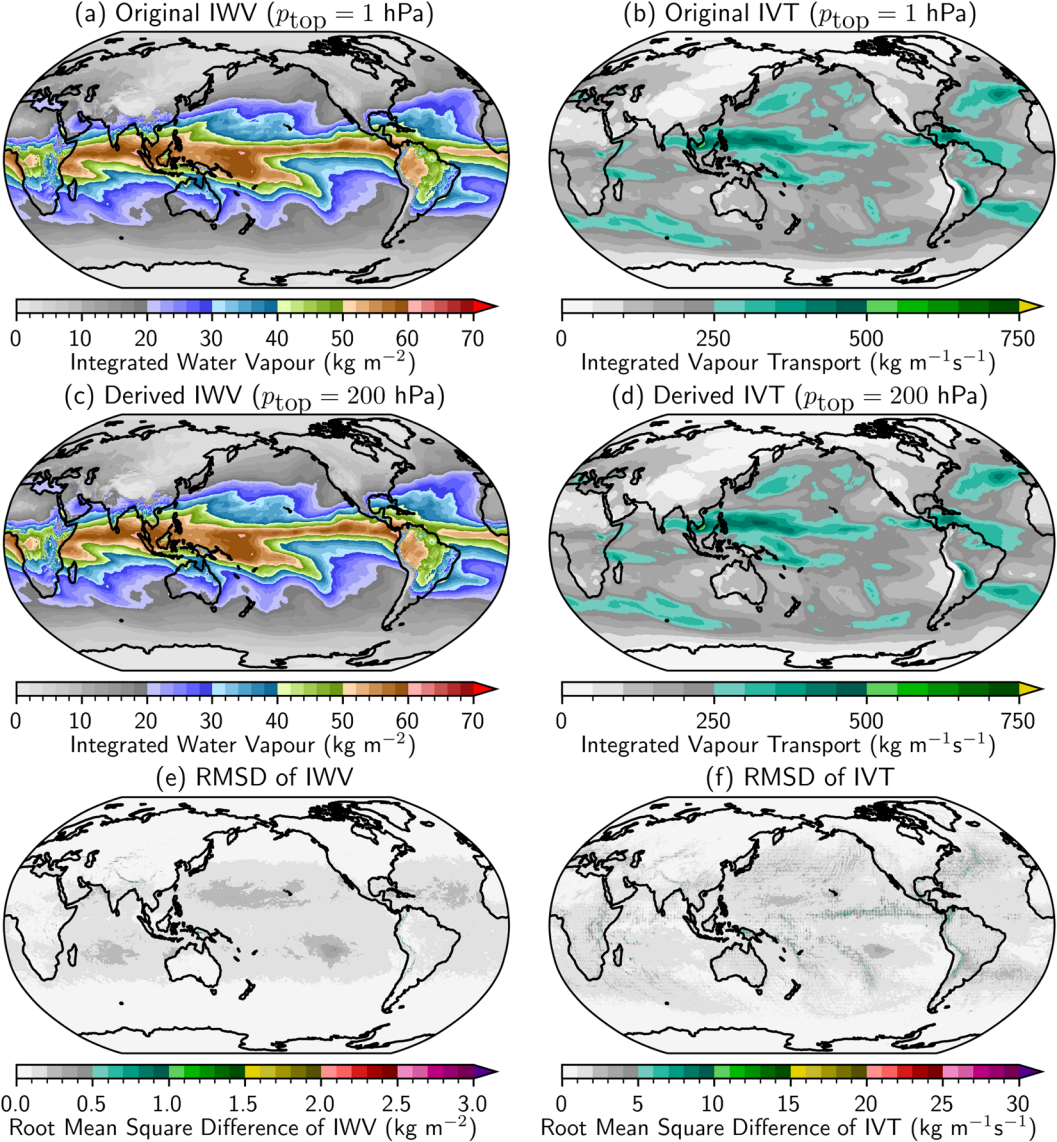
Global distributions of monthly mean IWV and IVT in November 2023. (**a,b**) The original IWV and IVT extracted from ERA5 based on vertical integration from the surface up to 1 hPa. (**c,d**) The derived IWV and IVT in EDARA based on vertical integration to 200 hPa. (**e**) The root mean square difference (RMSD) between the 6-hourly original and derived IWV. (**f**) The RMSD between the original and derived IVT.

As mentioned in Methods section, in accordance with common practice in AR analysis, the variables IWV, IVT (Qu, Qv), and CRH in EDARA are based on vertical integration from the surface up to the 200 hPa pressure level. Note that IWV, Qu and Qv are equivalent to the variables in ERA5 named total column water vapour, vertical integrals of eastward and northward water vapour flux, respectively. These ERA5 variables are derived from vertical integration up to the 1 hPa pressure level. The differences between these two integration limits should be very small because specific humidity decreases rapidly with height^60^. Figure 8 shows the monthly mean distributions of the original (ERA5) and re-derived (EDARA) IWV and IVT in November 2023. The differences between them are visually indistinguishable. The monthly mean IWV differences are in the range from −0.7 to 2.7 ${\rm{k}}{\rm{g}}\,{{\rm{m}}}^{-2}$, and the IVT differences are between −22.9 and 29.1 ${\rm{k}}{\rm{g}}\,{{\rm{m}}}^{-1}{{\rm{s}}}^{-1}$. The distributions of the root mean square differences of 6-hourly IWV and IVT are shown at the bottom row of Fig. 8. Their maximum values are only 2.8 ${\rm{k}}{\rm{g}}\,{{\rm{m}}}^{-2}$ and 38.9 ${\rm{k}}{\rm{g}}\,{{\rm{m}}}^{-1}{{\rm{s}}}^{-1}$, respectively.

The mtARget-v3 algorithm used to compute MARS in EDARA is slightly modified from the original tARget-v3 algorithm. By applying the direction requirement (poleward component of mean IVT > 50 kg m^−1^ s^−1^) only over the tropical region between 20°S and 20°N, mtARget-v3 can identify more extratropical AR objects than tARget-v3 does (see Fig. 6). At all 0000, 0600, 1200, 1800 UTC through July 1993, the total numbers of AR objects detected by mtARget-v3 (MARS) and tARget-v3 (ARS) are 1999 and 1607, respectively, corresponding to a ratio of 1.24.

## Usage Notes

**Fig. 9:**
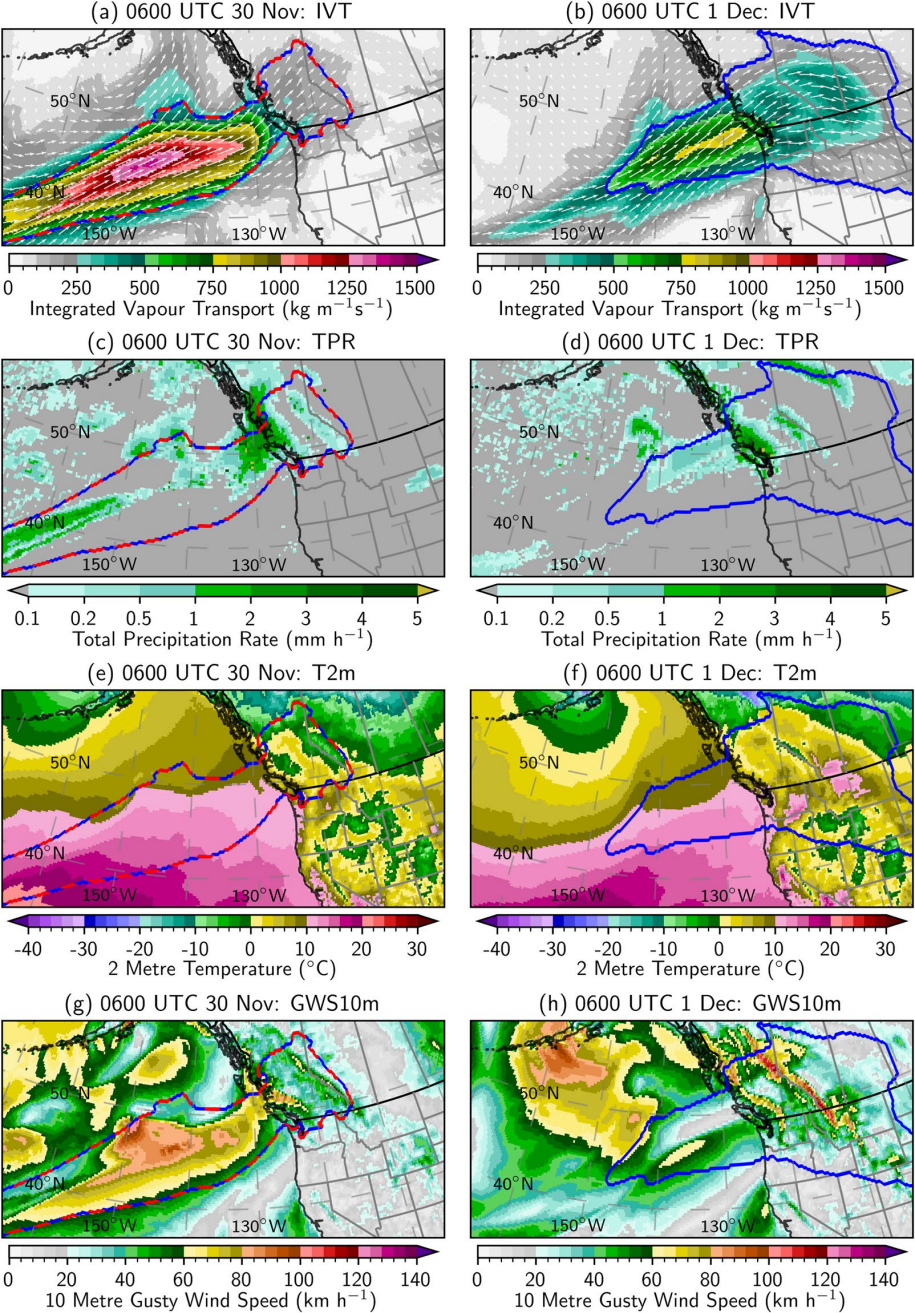
An atmospheric river analysis valid at 0600 UTC on 30 November (left panel) and 1 December (right panel) 2021. (**a,b**) IVT (Qu, Qv) distributions. (**c,d**) TPR distributions. (**e,f**) T2m distributions. (**g,h**) GWS10m distributions.

This dataset can be used to perform various types of AR analyses, such as severe weather case studies, model verification, climate change detection, and development of machine learning applications. As an example, Fig. 9 shows a case study using the EDARA variables of IVT (Qu, Qv), TPR, T2m, GWS10m, ARS, and MARS to investigate an AR-induced Chinook (föhn) event in western North America. The patterns of precipitation, temperature and wind distributions are consistent with the rapid development of warm, dry, and strong winds on the lee sides of the Coast Mountains and the Rocky Mountains when an AR made landfall and penetrated deep into the continent. There is observational evidence indicating that this event brought wind gusts of nearly 200 ${\rm{k}}{\rm{m}}\,{{\rm{h}}}^{-1}$ to parts of Alberta, Canada^73^.

To reduce the cost of archive storage, EDARA can only provide 12 AR-relevant variables at a 6-hour time step. For some comprehensive studies, users may need to download extra hourly data at multiple levels from ERA5 or other reanalysis suites. To access the graphical catalogues with a web browser, users may experience a freezing interface for a few seconds when the browser preloads all the images of a month to cache. Individual image can be downloaded by right-clicking and selecting the save method.

The ARS and MARS variables in EDARA are derived from the IVT distribution using the tARget-v3 and mtARget-v3 algorithms, respectively. The associated AR objects may not always coincide with their impacts, such as the areas of AR-induced heavy precipitation. It is well known that precipitation is associated with net convergence of water vapor flux rather than with the intensity of moisture transfer^60^. As shown in Fig. 4, some areas of high TPR in China are located to the northwest of the AR, where the moisture flux (Qu, Qv) converges to some local maximum values. For the 2008 case (Fig. 7), on the other hand, the band of maximum TPR is well located within the AR profile over southeastern China. Note that users can calculate the integrated moisture convergence with Qu and Qv included in EDARA.

Note that the TPR, TP6H and GWS10m fields are not available for 0000 and 0600 UTC 1 January 1940. This problem occurs because these variables in ERA5 are forecast parameters and the first ERA5 forecast was initiated at 0600 UTC 1 January 1940^39^.

EDARA should inherit the same issues of some temporal inhomogeneities and systematic biases identified in ERA5^37,42,65,74^. For the years from 2000 to 2006, the original ERA5 analyses of lower stratospheric temperature exhibit a pronounced cold bias due to specifying inappropriate background error covariances for the data assimilation^75^. To address this problem, ECMWF had produced a new set of analyses (termed ERA5.1) for this 7-year period. The EDARA variables over this period were derived from ERA5.1. Also note that ERA5 data are informed/constrained by observations through data assimilation, but that the degree of observational constraint differs by variable. For near-surface variables like TPR, TP6H and GWS10m, there may be notable discrepancies between ERA5 and “true” observations, even discounting scale mismatch.

## Data Availability

The codes used to generate and read the dataset are available in the misc folder at 10.20383/103.0935^43^.
